# Supplementary material for: Splice-Junction-Based Mapping of Alternative Isoforms in the Human Proteome
Source: Cell Rep. Author manuscript; Available in PMC 2020 Jan 15. (PMC6961840; doi:10.1016/j.celrep.2019.11.026)

A

sp|A0PJZ3|GXLT2_HUMAN|ENSG00000172986|MXE1|1768|chr3|72922335|72955349|+0|r17|T2
LSMKVCLFCMETEASTM[15.99]TISNQRSEHSMK q value: 0.0015649 Tr_novel:TRUE RefSeq_Novel:FALSE
Search result spec prec mz: 864.1448 Actual spec prec mz: 864.14478
Fragments matched per AA: 1.28 Proportion of top 20 peaks matched: 0.25

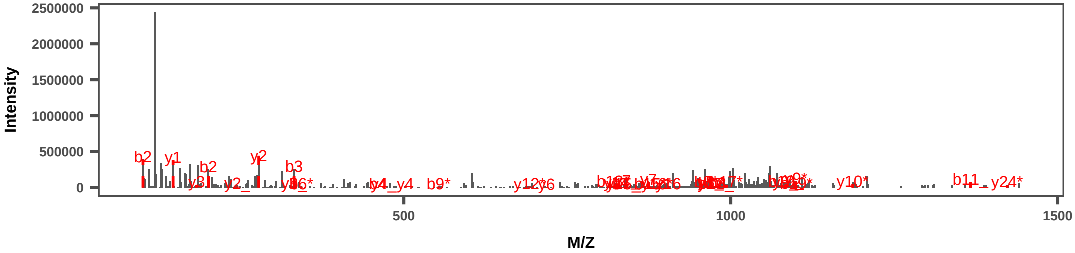

B

Scatterplot of predicted elution time
Fitting R2: 0.878
Novel peptide residual Z score: 1.23
Number of peptides: 6136

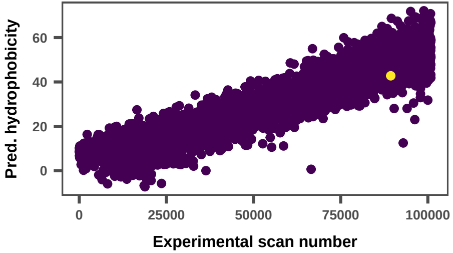

C

Distributions of residuals from best−fit line
of predicted RT vs Expt. scan number
Line: Z score of novel peptide
Z: 1.23

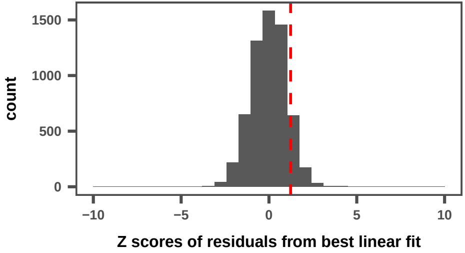

Supplement: 2 [file NIHMS1546469-supplement-2.zip › DF1/PXD006675/SmoothMuscleCells/SmoothMuscleCells_7_GXYLT2_LSMKVCLFCMETEASTMTISNQRSEHSMK.pdf]
